# Marker-dependent associations among oxidative stress, growth and survival during early life in a wild mammal

**DOI:** 10.1098/rspb.2016.1407

**Published:** 2016-10-12

**Authors:** Louise L. Christensen, Colin Selman, Jonathan D. Blount, Jill G. Pilkington, Kathryn A. Watt, Josephine M. Pemberton, Jane M. Reid, Daniel H. Nussey

**Affiliations:** 1 Institute of Biological and Environmental Sciences, School of Biological Sciences, University of Aberdeen, Aberdeen AB24 2TZ, UK; 2 Glasgow Ageing Research Network (GARNER), Institute of Biodiversity, Animal Health and Comparative Medicine, University of Glasgow, Glasgow AB24 2TZ, UK; 3 College of Life and Environmental Sciences, University of Exeter, Penryn Campus, TR10 9EZ, UK; 4 Institute of Evolutionary Biology, University of Edinburgh, Edinburgh EH9 3JG, UK

**Keywords:** antioxidants, early life fitness, life-history trade-offs, oxidative damage, plasma, Soay sheep

## Abstract

Oxidative stress (OS) is hypothesized to be a key physiological mechanism mediating life-history trade-offs, but evidence from wild populations experiencing natural environmental variation is limited. We tested the hypotheses that increased early life growth rate increases OS, and that increased OS reduces first-winter survival, in wild Soay sheep (*Ovis aries*) lambs. We measured growth rate and first-winter survival for four consecutive cohorts, and measured two markers of oxidative damage (malondialdehyde (MDA), protein carbonyls (PC)) and two markers of antioxidant (AOX) protection (total AOX capacity (TAC), superoxide dismutase (SOD)) from blood samples. Faster lamb growth was weakly associated with increased MDA, but not associated with variation in the other three markers. Lambs with higher SOD activity were more likely to survive their first winter, as were male but not female lambs with lower PC concentrations. Survival did not vary with MDA or total TAC. Key predictions relating OS to growth and survival were therefore supported in some OS markers, but not others. This suggests that different markers capture different aspects of the complex relationships between individual oxidative state, physiology and fitness, and that overarching hypotheses relating OS to life-history variation cannot be supported or refuted by studying individual markers.

## 1. Introduction

Oxidative stress (OS) has been proposed as a potential mediator of life-history trade-offs, providing a mechanistic explanation for variation in individual resource partitioning between self-maintenance, reproduction and growth [1,2]. Reactive oxygen species (ROS) are primarily by-products of normal aerobic metabolism [3]. Although ROS play crucial roles in cell-signalling and immune function [4], excess ROS can cause damage to macromolecules, such as lipids, proteins and DNA, and disrupt normal cellular function [5–7]. The state of imbalance between the dietary and endogenous antioxidant (AOX) defences and excess ROS, in favour of the latter, is known as OS [8].

Investment in rapid growth during early life could confer substantial individual fitness benefits, such as predator avoidance, improved competitive ability, enhanced thermoregulation, and hence increased survival probability [9]. However, growth is highly physiologically demanding, and rapid growth may be accompanied by increased metabolic rate and thus oxygen consumption. Therefore, individuals that grow faster may be exposed to relatively more ROS ([6,10] although see [11]), and increased investment in growth may limit resources available for AOX defences [12]. Consequently, faster-growing individuals are predicted to experience greater OS, which may ultimately impair physiological function and reduce survival probability and fitness [1]. OS could therefore provide a causal explanation for covariation in growth rates and survival, thereby shaping resource allocation, and ultimately life-history evolution. However, to date, few studies have simultaneously tested the two hypotheses that faster growth during early life is associated with OS, and that OS is associated with subsequent survival, within wild populations experiencing natural environmental variation that might constrain growth and survival. Further, despite considerable research, the degree to which OS might generally influence early life growth and survival, or *vice versa*, remains unclear.

Some previous studies spanning captive [13–16] and wild [10,17–19] vertebrates and invertebrates found no correlations between growth rate and OS markers [20], while other studies found positive correlations between growth and oxidative damage [10,13,16–18,21,22]. There is also evidence for both negative [14] and positive correlations between growth and AOX [15,19,21]. Similarly, no overarching consensus regarding links between OS and survival has yet emerged (summarized by [23]). OS predicted survival in some systems (e.g. [24]), but not in others (e.g. [23,25]). Further, most such studies have been conducted in captive populations under laboratory conditions. Laboratory studies allow environmental and genetic variation to be tightly controlled, and can experimentally manipulate growth or OS and sample and analyse OS across multiple occasions and tissues to examine whether effects are repeatable and tissue-specific or organism-wide [26]. However, both the degree of OS experienced and its fitness consequences are likely to be highly dependent on environmental conditions. Natural populations typically experience more variable and physiologically challenging environments than laboratory populations, and might consequently show different relationships between growth, OS and survival. Therefore, although wild population studies may not readily allow experimentation or repeated sampling, they form a crucial part of our overall endeavour to understand the evolutionary causes and consequence of variation in OS [2]. Further, since environmental conditions and associated constraints on life-history allocation commonly vary among seasons and years, datasets quantifying variation in growth, OS and survival across multiple different years are ideally required to fully understand the role of physiological trade-offs in shaping life-history evolution. However, multi-year studies investigating the associations between growth and OS, and OS and survival in wild populations remain rare.

We used life-history data and plasma samples collected during four consecutive summers from a sexually dimorphic wild Soay sheep (*Ovis aries*) population inhabiting a naturally variable environment to quantify associations between four commonly applied markers of OS and early life growth and survival. Specifically, we used OS marker data collected on lambs to test the hypotheses that: (i) increased growth rate during early life carries an oxidative cost and is therefore positively correlated with oxidative damage and negatively correlated with AOX and (ii) independent of growth rate, increased oxidative damage and low levels of AOX protection reduce a lamb's first-winter survival probability. We tested whether associations between OS, growth and survival were consistent across different markers, across females and males and across 4 years spanning different environmental conditions. We thereby quantify the magnitude and heterogeneity of associations between major physiological and life-history traits that could underlie key life-history trade-offs.

## 2. Material and methods

### (a) Study system and sample collection

Plasma samples were collected from Soay sheep, resident in Village Bay, Hirta, in the St Kilda archipelago (57°49′ N, 8°34′ W). Since 1985, the population has been monitored intensively. Pregnant ewes are monitored closely prior to and during lambing. Lambs are caught, uniquely ear-tagged and weighed a few days after birth, between mid-April and early May. Each lamb's sex is determined by visual examination; a scrotum sack is visible in males. The identity of each lamb's mother, and the lamb's twin status (i.e. twin or singleton), is recorded. Lambing is followed by an annual August catch where sheep are rounded up and caught in temporary traps. Individuals are identified from their tag, weighed, measured and blood sampled. Regular censuses throughout the year and mortality searches through winter and spring mean that the vast majority of carcasses are found and accurate survival data are available [27].

We used blood samples collected from lambs during four consecutive Augusts (2010–2013), to quantify OS. Blood was collected in 9 ml lithium heparin vacutainers by venepuncture and stored at 4°C until processing the following day. Whole blood was spun for 10 min at 1008 g, and the plasma supernatant was collected and stored at −80°C until further use. Samples were transported from St Kilda using either liquid nitrogen vapour shippers (2010 and 2011) or a portable −80°C freezer (2012 and 2013: Stirling Shuttle Ultra Low Portable Freezer. Triple Red Laboratory Technology, Bucks, England).

### (b) Measures of oxidative stress

We measured two commonly applied markers of oxidative damage: protein carbonyls (PC), a measure of protein damage and malondialdehyde (MDA), a measure of lipid peroxidation. We also measured two markers of AOX protection: superoxide dismutase (SOD), an endogenously produced AOX enzyme, and total antioxidant capacity (TAC), a combined measure of several AOXs, both endogenous and dietary-derived. Full assay protocols are provided in electronic supplementary material, appendix S1 (see also [28]). In brief, plasma PC content (nmol mg^–1^ protein) was quantified using a Cayman Protein Carbonyl Assay Kit (ID:10 005 020; Cayman Chemical Company, Ann Arbour, MI, USA). Protein content was quantified using the Bradford method [29]. Plasma MDA (µM l^−1^) was quantified using high-performance liquid chromatography, following [17]. Total plasma SOD activity (U ml^−1^) was quantified using Cayman's Superoxide Dismutase Assay Kit (ID: 706 002; Cayman Chemical Company) following the manufacturer's protocol. Plasma TAC levels (mM) were estimated using Cayman's Antioxidant Assay Kit (ID: 709 001; Cayman Chemical Company). These standard assays return high within-sample repeatabilities and low inter-plate coefficients of variation [28]. The four markers were uncorrelated within years across individuals included in current analysis (electronic supplementary material, table S1, see also [28]).

We also previously measured MDA from plasma samples collected in August 2007, using identical methods [17]. This initial single-year single-marker study showed a positive association between MDA and lamb growth rate, indicating an oxidative damage cost of fast growth [17]. It has recently been suggested that MDA levels are positively related to circulating triglyceride levels in birds [30], but owing to the uncertain causes of this relationship we did not attempt to correct for plasma lipid content in this study.

### (c) Statistical analysis

To test the hypothesis that rapid growth increases OS, we used 236 lambs for which we had data on all four OS markers, sex, twin status, April (birth) weight, August weight and maternal identity (2010 *n* = 32, 17♀ and 15♂; 2011 *n* = 76, 38♀ and 38♂; 2012 *n* = 43, 24♀ and 19♂ and 2013 *n* = 85, 41♀ and 44♂). A lamb's growth rate over approximately its first four months of life was calculated by dividing the difference in lamb mass at August capture and April capture by the number of days elapsed between the two capture events (following [17]). Note also that August weight and growth rate were strongly correlated in this dataset; Pearson product moment correlation, *p* < 0.01, *r* = 0.97, d.f. = 234). We fitted separate linear mixed models (LMMs) with each OS marker as the dependent variable. We fitted main fixed effects of year (4-level factor) and an individual lamb's sex (2-level factor), twin status (2-level factor) and growth rate to assess if these explained variation in marker values. Since year, sex and twin status can all influence growth rate (e.g. [17]), first-order interactions between growth rate and year, growth rate and sex and growth rate and twin status were also modelled. We also included random maternal identity effects to account for non-independence of multiple lambs from the same mother. Since we also had MDA and growth data from 84 lambs sampled and measured in August 2007 [17], we refitted the LMM for MDA including these data.

To test the hypothesis that OS predicts first-winter survival, we restricted statistical analyses to samples from lambs born in 2010 and 2013 when overall first-winter mortality was 65.9% and 72.3%, respectively. Samples from lambs born in 2011 and 2012 had to be excluded because first-winter mortality was very high (93.8%) and very low (4.0%) in these years, respectively, meaning there was very little variation in survival to explain (see §4). We included 135 lambs for which we had data on all four markers of OS: first-winter survival, August weight, sex and maternal identity (2010 *n* = 41, 25♀ and 16♂ and 2013 *n* = 94, 43♀ and 51♂). We fitted a generalized linear mixed model (GLMM) with survival as a binary-dependent variable (0 = died, 1 = survived) with binomial error structure and logit link. We fitted all four OS markers as covariates within a single model, along with main effects of year (2-level factor) and sex (2-level factor), and the first-order interactions between each marker and year and sex. We also fitted August weight as a covariate, and fitted random maternal identity effects.

Fixed effects in LMMs and GLMMs fitted with maximum likelihood were simplified following a backwards elimination approach, using likelihood ratio tests. Terms with the lowest marginal *χ*^2^-statistic were sequentially dropped until only significant (*p* < 0.05) explanatory variables remained. Main effects underlying retained interaction terms were also retained. All analyses were run in R v. 2.15.2 [31].

## 3. Results

### (a) Oxidative stress and growth rate

**Figure 1.**
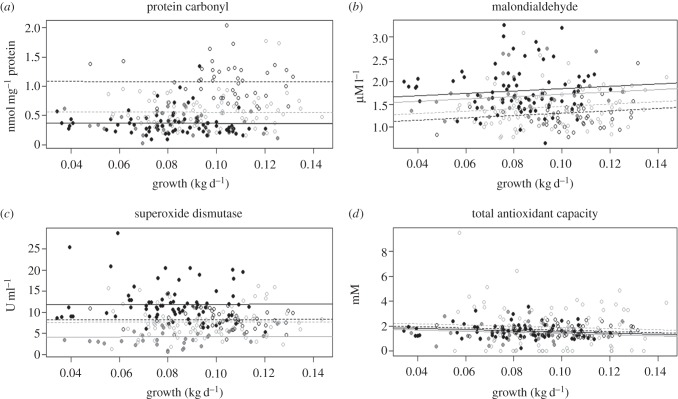
Relationships between four markers of OS markers and growth rate (increase in mass over time, kg d^−1^) in Soay sheep lambs, with year (2010 = grey solid dots, 2011 = black solid dots, 2012 = black open dots and 2013 = grey open dots, total *n* = 236). (*a*) Protein carbonyl (PC, nmol mg^−1^ protein), (*b*) malondialdehyde (MDA) (µM l^−1^), (*c*) superoxide dismutase (SOD) (U ml^−1^) and (*d*) total antioxidant capacity (TAC) (mM). Lines show predicted marker levels by year (2010 = grey line, 2011 = black line, 2012 = black dotted line and 2013 = grey dotted line).

We found a marginally non-significant positive association between MDA and lamb growth rate across lambs sampled during 2010–2013, but no indication of interactions between growth and year, sex or twin status (table 1 and electronic supplementary material, table S2; figure 1). When we refitted our model including the additional MDA data from 2007, we estimated similar effect sizes but the overall effect of growth rate on MDA was marginally significant (estimated slopes of 2.62 ± 1.49 s.e. excluding 2007 and 2.58 ± 1.17 s.e. including 2007; electronic supplementary material, table S3). Overall, these models suggest that, as predicted, there is a positive association between MDA and growth rate, but that this association is weak and of marginal statistical significance (figure 1 and table 1; electronic supplementary material, table S3). Our models for PC, TAC and SOD showed no significant associations with growth rate, or interactions between growth rate and year, sex or twin status (table 1 and figure 1; electronic supplementary material, table S2). Furthermore, none of the four markers differed significantly between male and female lambs, or between singletons versus twin lambs (table 1). However, PC, MDA and SOD, but not TAC, varied significantly among years (table 1; electronic supplementary material, figure S1). PC concentration was highest in 2012, MDA content was lower in 2012 and 2013 than in 2010 and 2011 (and also low in 2007), and SOD activity was lowest in 2010 and highest in 2011 (electronic supplementary material, figure S1).

**Table 1.** Linear mixed models of four different biomarkers of OS in response to growth rate (kg d^−1^) in Soay sheep lambs and associated degrees of freedom (d.f.), estimates and standard errors, for (*a*) protein carbonyls, (*b*) malondialdehyde, (*c*) superoxide dismutase and (*d*) total antioxidant capacity. Intercepts were set to female lambs in 2010. All models show terms retained after model simplification, along with dropped main effects in order of elimination (full models, including interactions and random effects, are shown in electronic supplementary material, table S2). All models used data from 236 lambs collected in 2010–2013. Italics indicate statistical significance at or below threshold 0.05.

term	d.f.	*χ*^2^	*p*-value	fixed effects	estimate	s.e.
(*a*) protein carbonyls						
final model (conditional *R*^2^ = 0.51)						
year	*3*	*170*.*98*	*<0*.*001*	intercept	0.37	0.05
				2011	−0.005	0.05
				2012	0.71	0.06
				2013	0.19	0.05
dropped terms						
twin	1	0.51	0.47		−0.04	0.05
growth	1	0.19	0.66		−0.40	0.92
sex	1	0.67	0.41		0.03	0.05
(*b*) malondialdehyde						
final model (conditional *R*^2^ = 0.44)						
year	*3*	*49*.*07*	*<0*.*001*	intercept	1.68	0.08
				2011	0.12	0.09
				2012	−0.38	0.10
				2013	−0.25	0.09
dropped terms						
sex	1	<0.01	0.99		<−0.01	0.06
twin	1	0.49	0.49		−0.06	0.09
growth	1	3.05	0.08		2.62	1.49
(c) superoxide dismutase						
final model (conditional *R*^2^ = 0.64)						
year	*3*	*108*.*79*	*<0*.*001*	intercept	4.23	0.61
				2011	7.65	0.71
				2012	4.07	0.76
				2013	3.49	0.70
dropped terms						
growth	1	0.17	0.68		−5.73	13.91
sex	1	0.12	0.73		0.14	0.41
twin	1	0.55	0.46		−0.44	0.60
(*d*) total antioxidant capacity						
final model						
* none*						
dropped terms						
sex	1	0.03	0.87		0.03	0.16
twin	1	0.57	0.45		−0.16	0.22
growth	1	1.62	0.20		−4.77	3.75
year	3	6.26	0.10			
2011					0.14	0.24
2012					0.17	0.26
2013					0.44	0.23

### (b) Oxidative stress and survival

**Figure 2.**
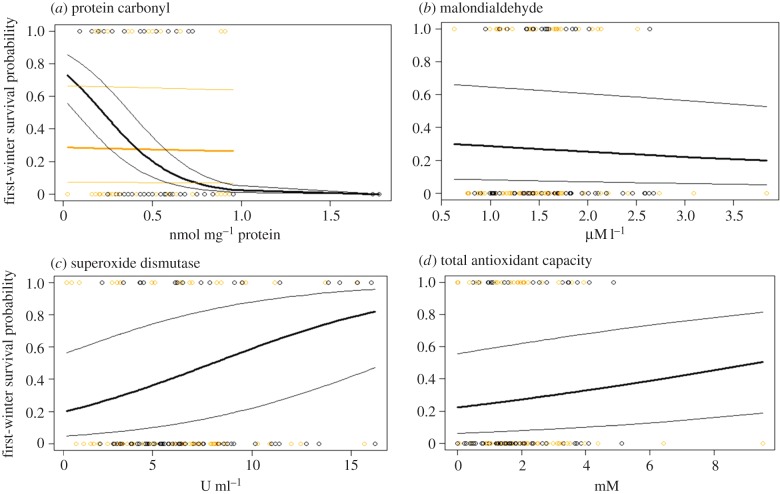
Relationships between observed first-winter survival (1: survived, 0: died) and predicted survival probabilities for Soay sheep lambs and (*a*) PC (nmol mg^−1^ protein), (*b*) MDA (µM l^−1^), (*c*) SOD (U ml^−1^) and (*d*) TAC (mM), respectively. Thick and thin lines show predicted survival ±1 s .e. Males (black) and females (orange) of all sampled lambs of 2010 and 2013 (*n* = 135). (Online version in colour.)

The final model for lamb first-winter survival included main effects of SOD and August weight, and a PC-by-sex interaction (table 2). Lambs with higher SOD activity in August were more likely to survive their first winter (table 2 and figure 2*c*). Independent of this association, male lambs with high PC concentration were less likely to survive their first winter than male lambs with low PC concentrations, whereas the probability of female lamb survival did not vary with PC concentration (table 2 and figure 2*a*). The PC-by-sex interaction remained significant when two male lambs with particularly high PC concentrations were excluded from the analysis (d.f. = 1, *χ*^2^ = 5.44, *p* = 0.02, *n* = 133). MDA and TAC did not predict survival either as main effects or through interactions with year or sex (table 2 and electronic supplementary material, table S4). As expected, lambs that were heavier in August were more likely to survive their first winter (table 2).

**Table 2.** Generalized linear mixed model of first winter survival in relation to four different biomarkers of OS (PC, MDA, SOD and TAC) in Soay sheep lambs and associated degrees of freedom (d.f.), estimates and standard errors. The intercept was set to female lambs in 2010. Model terms retained after simplification are shown, along with dropped main effects in order of elimination (the full model, including interactions and random effects, is shown in electronic supplementary material, table S4). The model used data from 135 lambs collected in 2010 and 2013. Italics indicate statistical significance at or below threshold 0.05.

term	d.f.	*χ*^2^	*p*-value	fixed effects	estimate	s.e.
survival						
final model (conditional *R*^2^ = 0.54)				intercept	−6.82	2.63
SOD	*1*	*4*.*30*	*0*.*04*	SOD	0.16	0.09
August weight	*1*	*15*.*32*	*<0*.*001*	August weight	0.42	0.17
PC × sex	*1*	*5*.*74*	*0*.*02*	PC × sex	−6.02	3.12
sex				sex	1.24	1.30
PC				PC	−0.23	1.56
dropped terms						
MDA	1	1.04	0.31		−0.56	0.52
TAC	1	0.98	0.32		0.17	0.18
year	1	1.66	0.20		−0.83	0.70

## 4. Discussion

We tested whether relationships consistent with trade-offs between OS and growth rate were evident in Soay lambs, and tested whether OS predicted first-winter survival, a key fitness component. Moreover, we tested whether relationships were consistent across four OS markers encapsulating oxidative damage and AOX protection, across four different years, and across females and males. We found some evidence of the predicted positive association between MDA and growth rate, but the estimated effect was only marginally statistically significant. The other three markers, PC, SOD and TAC, did not vary significantly with growth rate. Lamb first-winter survival showed the predicted positive association with SOD, and the predicted negative association with PC in male lambs but not in females, independent of August weight. However, survival did not vary with MDA or TAC. Therefore, overall, our results provide only partial and somewhat inconsistent support for the key hypotheses that rapid growth increases OS, and that increased OS reduces survival probability.

Some previous studies in wild birds reported the predicted negative associations between AOX levels and growth [10,18,32], but a recent meta-analysis of both wild and captive studies found evidence for an oxidative cost of increased growth only in markers of oxidative damage and not in measures of AOX [33]. The authors suggested this could reflect the inherent complexity of the AOX response to OS, with antioxidants potentially being differentially upregulated and depleted through utilization during growth [33]. Although our study adds to the growing literature documenting positive associations between markers of oxidative damage and growth [17,18,21], it is perhaps surprising that we only found a weak association between growth and MDA, and no association with PC. However, a study of captive domestic lambs (breed: Ile de France x INRA 401) also found no association between experimentally induced compensatory growth and PC [34]. Surprisingly, since growth rate and August weight are both strong predictors of lamb survival on St Kilda [35], PC but not MDA predicted lamb over-winter survival. This suggests that the high MDA levels associated with faster growth have little impact on subsequent survival, and that variation in PC reflects a different aspect of oxidative state that affects survival, at least in males. Overall, therefore, our results highlight the value of relating OS to both growth and fitness components (such as survival) in natural populations; solely relating OS to growth would have led to incorrect inferences regarding the fitness consequences of variation in different markers of oxidative damage.

Indeed, our study represents a rare test of associations between OS and survival in a wild mammal. We observed substantial among-year variation in three of our four OS markers, and in lamb first-winter survival. Indeed, in 2011 and 2012 lamb mortality was so high and low, respectively, that within-year variation could not be analysed. In these years, it is self-evident that among-lamb variation in OS does not drive variation in first-winter survival. This is not surprising, as extremely high or low first-winter survival on St Kilda is known to be largely driven by extrinsic factors including population density, winter climate and gastro-intestinal nematode parasite burdens [27]. Further, the high and low mortality winters were not associated with extreme mean oxidative damage or AOX values (electronic supplementary material, figure S1). For instance, mean PC was highest in 2012 when lamb mortality was low (4.0%) and mean SOD was highest in 2011 when lamb mortality was very high (93.8%). However, associations between lamb PC and SOD and first-winter survival within the two moderate mortality years (2010 and 2013) suggests that these markers may reflect aspects of the lambs' physiological state that are associated with their ability to survive winters when malnutrition, winter weather and parasite pressures are not too severe.

Our finding that lambs with high SOD were more likely to survive their first winter in moderate mortality years matches the prediction that higher investment in AOX should protect the individual from damage and promote physiological function and survival. However, it raises the question of why SOD matches the prediction while the other markers do not. Whereas our TAC assay measures the combined effect of a suite of endogenous and exogenous AOX, our SOD assay measures a specific group of endogenously produced AOX. Variation in SOD levels in blood or tissue might therefore be a more repeatable measure of an individual's intrinsic ability to resist OS, and may be more likely to reflect the oxidative state of a lamb several months later at the onset of winter. Most other studies examining associations between OS markers (measured in blood) and survival in wild populations come from birds, and findings are quite mixed [23]. For example, low plasma MDA was associated with higher adult recruitment in European shag (*Phalacrocorax aristotelis*) nestlings [36]. King penguin (*Aptenodytes patagonicus*) chicks that did not survive the initial growth period had the lowest plasma AOX capacities and highest oxidative damage [10], although this study did not directly analyse effects of OS on survival. In one population of great tits (*Parus major*), SOD activity measured in red blood cells (RBCs) was not associated with nestling survival [37], while in another population individuals that expressed intermediate activity of an important AOX enzyme, glutathione peroxidase, had highest survival probability [38]. In one of few other studies of wild mammals, MDA, but not PC or SOD, was associated with survival in adult wild mongoose (*Mungos mungo*) [39].

Further, associations between PC and survival were sex-specific in Soay lambs; although mean PC values did not differ between females and males, high PC was associated with reduced first winter survival probability in males but not females. Some other studies have reported sex-specific marker values, and/or sex-specific associations with survival. In rats (*Rattus norvegicus*), protein hydroperoxide, a cause of protein oxidation, was higher in males than in same-age females, but PC content did not differ significantly [40]. Furthermore, a sex-dependent association between OS and survival was found in alpine swifts (*Apus melba*), where RBC resistance to oxidation was higher in adult males but not females that returned the following year [41]. Soay sheep are highly polygynous and sexually dimorphic. Male lambs, under the influence of androgens, undergo more rapid growth than females, develop secondary sexual characters and are sexually mature and capable of siring offspring (although they rarely do) in the November rut in their birth year [42]. Male lambs are also less likely to survive their first winter than female lambs, at least partly owing to their investment in growth and sexual development [27]. Such early life investment in growth and reproduction might be reflected in PC values, because of the associated increase in ROS production and protein damage. This variation may predict survival in males but not females because males pay higher survival costs of growth and reproduction in their first year. Indeed, a review of human and rat studies investigating sex differences in OS found that regardless of whether male or female rats have higher OS levels in various tissues, males are ultimately more prone to OS-modulated blood pressure effects than females [43]. Thus, males might be overall more sensitive to OS than females, possibly owing to the AOX effects of oestrogen [44]. Although speculative, this hypothesis could be tested by quantifying associations between PC and reproductive investment traits, and thereby testing whether oxidative costs of reproduction are sex-dependent.

Overall, our current study, alongside other studies on wild vertebrates, suggests that associations between OS markers, growth and survival are highly marker- and context-dependent. A fundamental challenge facing evolutionary and physiological ecologists aiming to understand the role of OS in mediating life-history trade-offs is therefore to understand which OS markers behave as currently predicted by life-history theory and when they do so, and which markers do not. Possible explanations are that physiological processes associated with growth, reproduction and survival elicit certain types of oxidative damage and AOX but not others [39], or that different types of OS are differentially affected by independent biotic or abiotic factors that also affect growth and survival. Another possibility results from the fact that most wild population studies, including ours, are constrained to sample blood, and to sample individuals at single logistically feasible time points that may not coincide with expression of key life-history traits. Testing relationships between marker values and life-history traits therefore requires that blood-based OS markers reflect organism-wide oxidative state, and that individual measures are repeatable over time. However, some laboratory studies suggest that concentrations of particular OS markers may be only weakly correlated across tissues [43,45,46] and that some OS markers show circadian variation [47]. Yet, to date, studies that considered multiple tissues have all been cross-sectional, and longitudinal studies of OS markers measured in blood rarely report the within-individual repeatability (although see [48]). Longitudinal field and laboratory studies that quantify the repeatability of different OS markers across tissues and individuals over different times and life-history stages are challenging to implement, but are urgently required if we are to understand the role of OS in mediating life-history evolution.

## Supplementary Material

Appendix S1

## Supplementary Material

Figure S1

## Supplementary Material

Appendix Table S1

## Supplementary Material

Appendix Table S2

## Supplementary Material

Appendix Table S3

## Supplementary Material

Appendix Table S4

## Data accessibility

Data available from the Dryad Digital Repository: http://dx.doi.org/10.5061/dryad.s1d25 [49].
