# Supplementary material for: Marker-dependent associations among oxidative stress, growth and survival during early life in a wild mammal
Source: Proc Biol Sci. 2016 Oct 12;283(1840):20161407. doi: 10.1098/rspb.2016.1407 (PMC5069507; doi:10.1098/rspb.2016.1407)
Supplement: Appendix S1 [file rspb20161407supp1.docx]

ASSAY PROTOCOLS FOR MEASURES OF OXIDATIVE STRESS

Plasma PC content (nmol/ mg protein) was determined using a Cayman Protein Carbonyl Assay Kit (ID:10005020; Cayman Chemical Company, Ann Arbour, Michigan, USA). 2,4-dinitrophenyhydrazine (DNPH) was added to initiate a reaction with PCs leading to formation of a hydrazone detectable by spectrophotometry. We followed the manufacturer’s protocol except that during the 3x washing/resuspending of pellets with (1:1) ethanol/acetate, we added 500*µ*L ethanol/acetate per wash, rather than the specified 1mL. We measured absorbance at 370nm using a plate reader (Spectra Max Molecular Devices, Sunnyvale, CA, USA) and Softmax Pro 5.3 software. In order to determine the amount of PCs we standardized to protein concentration by dividing by protein content to give PC concentration (mg/mL). Protein content was determined following the Bradford method [27]. We used a Spectra Max plate reader (Molecular Devices, Sunnyvale. CA, USA) to measure absorbance at 595 nm.

Plasma MDA (*µ*M/L) was quantified using high-performance liquid chromatography (HPLC), following [17]. To ensure sufficient mixing and remove debris, samples were initially vortexed and centrifuged. 50 *µ*L of butylated hydroxytoluene, 400 *µ*L 0.44 M phosphoric acid solution, and 100 *µ*L (42mM) thiobarbituric acid (TBA) solution were added to 50 *µ*L of sample or standard (1,1,3,3-tetraethoxypropane, TEP). After vortexing for 5 seconds, samples were heated to 100ºC in a dry bath incubator. Samples were then cooled on ice for 5 minutes, spun down and 200 *µ*L *n*-butanol was added and mixed by vortexing for 20 seconds. Samples were then centrifuged at 15,338 g for 3 minutes at 4ºC and 90 *µ*L of the upper phase was collected and stored in a HPLC vial for analysis. 40 *µ*L of sample was injected into a HPLC-system (Dionex Corporation, Sunnyvale, CA, USA) fitted with a 5 *µ*m ODS 100 x 4.6 mm column oven maintained at 37ºC. A methanol buffer (40:60, 50mM anhydrous solution of potassium monobasic phosphate, pH 6.8) was running isocratically over 3.75 minutes (1 mL/minute) as the mobile phase. A fluorescence detector (RF2000; Dionex) set at 515 nm (excitation) and 553 (emission) recorded the data. A standard curve was prepared from a TEP stock solution, following serial dilution with 40% ethanol and used for calibration.

Total plasma SOD activity (U/mL) was determined using Cayman’s Superoxide Dismutase Assay Kit (ID: 706002; Cayman Chemical Company) following the manufacturer’s protocol. SOD activity was estimated by measuring the dismutation of superoxide radicals and calibrated against a bovine erythrocyte SOD (Cu/Zn) enzyme standard curve. Absorbance was read at 450 nm, and data were analysed using Softmax Pro 5.3 software. Samples from 2010 and 2011 were analysed using a Molecular Devices SpectraMax M2 plate reader, whereas samples from 2012 and 2013 were analysed using a SpectraMax plate reader.

Plasma TAC levels (mM) were estimated using Cayman’s Antioxidant Assay Kit (ID: 709001; Cayman Chemical Company). Following manufacturer’s protocol, a Trolox standard curve was used to quantitate the antioxidant capacity of the sample, measured in millimolar Trolox equivalents. Samples from 2010 and 2011 were analysed using a Molecular Devices SpectraMax M2 plate reader. Samples from 2012 were analysed using a SpectraMax plate reader and 2013 samples were analysed on a Thermo Scientific Multiscan GO plate reader. The absorbance was read at either 405 or 750 nm.
