# Supplementary material for: Marker-dependent associations among oxidative stress, growth and survival during early life in a wild mammal
Source: Proc Biol Sci. 2016 Oct 12;283(1840):20161407. doi: 10.1098/rspb.2016.1407 (PMC5069507; doi:10.1098/rspb.2016.1407)
Supplement: Figure S1 [file rspb20161407supp2.docx]

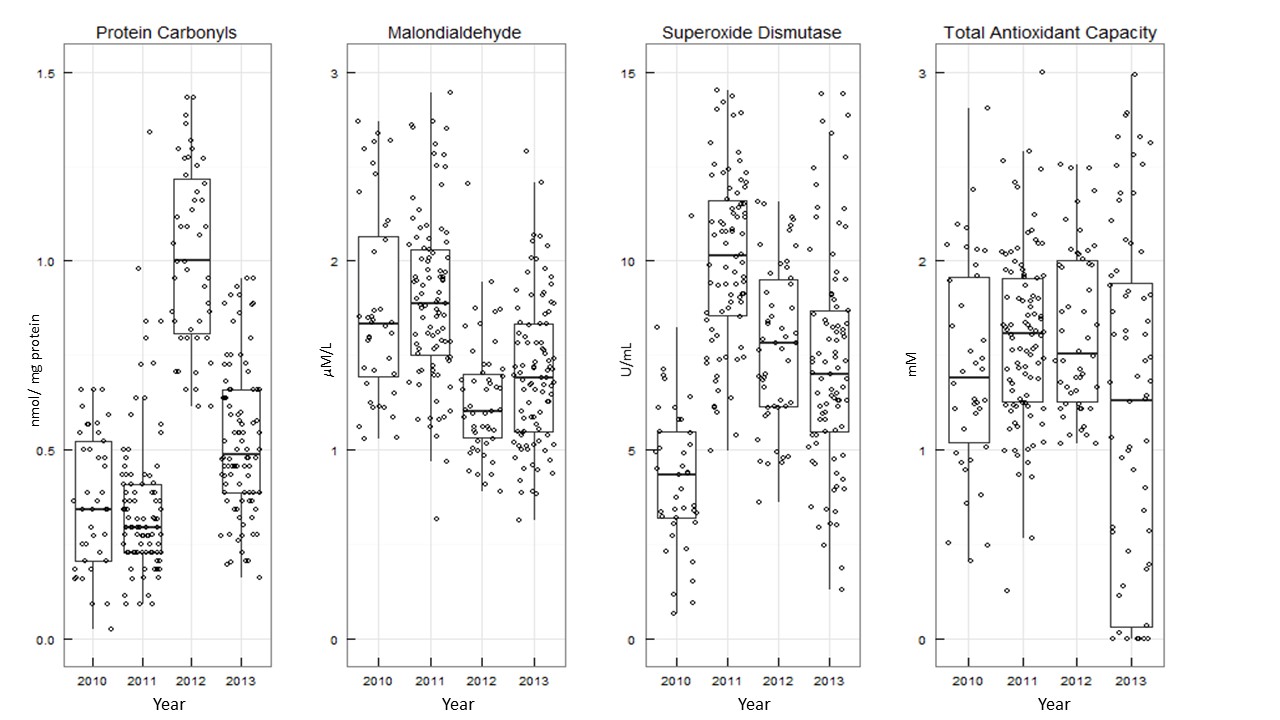


Figure S1. Among year variation in protein carbonyls (PC, nmol/ mg protein), malondialdehyde (MDA, *µ*M/L), superoxide dismutase (SOD, U/mL) and total antioxidant capacity (TAC, mM) measured in Soay sheep lambs born in 2010 (n= 33), 2011 (n= 77), 2012 (n= 43) and 2013 (n= 86). Bar and box show the median and interquartile range, and whiskers show the 10% and 90% quantiles. Dots show the raw data, jittered along the x-axis within each year for visualisation. For comparison, mean MDA content in 2007 was 1.32.
