# Supplementary material for: Marker-dependent associations among oxidative stress, growth and survival during early life in a wild mammal
Source: Proc Biol Sci. 2016 Oct 12;283(1840):20161407. doi: 10.1098/rspb.2016.1407 (PMC5069507; doi:10.1098/rspb.2016.1407)
Supplement: Appendix Table S1 [file rspb20161407supp3.docx]

| All lambs | | | | | | | | | | |
| --- | --- | --- | --- | --- | --- | --- | --- | --- | --- | --- |
| Marker/s | **All years** | | **2010** | | **2011** | | **2012** | | **2013** | |
|  | r | *P* | r | *P* | r | *P* | r | *P* | r | *P* |
| PC-MDA | -0.28 | **<0.001** | 0.24 | 0.19 | -0.17 | 0.14 | <0.01 | 1.00 | -0.04 | 0.73 |
| PC-SOD | -0.06 | 0.34 | -0.22 | 0.22 | 0.01 | 0.93 | -0.20 | 0.21 | 0.20 | **0.07** |
| PC-TAC | -0.02 | 0.83 | 0.27 | 0.13 | <0.01 | 0.97 | 0.33 | 0.03 | -0.15 | 0.19 |
| MDA-SOD | 0.21 | **0.002** | -0.12 | 0.51 | 0.17 | 0.14 | -0.05 | 0.77 | 0.13 | 0.23 |
| MDA-TAC | 0.03 | 0.66 | -0.12 | 0.53 | 0.01 | 0.94 | <0.01 | 0.99 | 0.15 | 0.17 |
| SOD-TAC | 0.02 | 0.80 | -0.15 | 0.43 | 0.21 | 0.06 | 0.04 | 0.81 | -0.01 | 0.91 |

**Table S1.** Pearson product-moment correlation coefficients (r) and P-values for the correlations between different markers of oxidative stress; protein carbonyl (PC), malondialdehyde (MDA), superoxide dismutase (SOD) and total antioxidant capacity (TAC).
