# Supplementary material for: Marker-dependent associations among oxidative stress, growth and survival during early life in a wild mammal
Source: Proc Biol Sci. 2016 Oct 12;283(1840):20161407. doi: 10.1098/rspb.2016.1407 (PMC5069507; doi:10.1098/rspb.2016.1407)
Supplement: Appendix Table S2 [file rspb20161407supp4.docx]

**Table S2.** Linear mixed models of four different biomarkers of oxidative stress in response to growth rate (kg day^-1^) in Soay sheep lambs and associated degrees of freedom (DF), estimates and standard errors, for (A) protein carbonyls (PC), (B) malondialdehyde (MDA), (C) superoxide dismutase (SOD) and (D) total antioxidant capacity (TAC). Intercepts were set to female lambs in 2010. All models show terms retained after model simplification, along with dropped terms in order of elimination. All models used data from 236 lambs collected in 2010-2013.

| Term | DF | X^2^ | p-value | Fixed effects | Estimate | Standard error |
| --- | --- | --- | --- | --- | --- | --- |
| **(A)** Protein carbonyls | | |  |  |  |  |
| Final model  (conditional R^2^= 0.51) | | | |  |  |  |
| Year | **3** | **170.98** | **<0.001** | Intercept | 0.37 | 0.05 |
|  |  |  |  | 2011 | -0.005 | 0.05 |
|  |  |  |  | 2012 | 0.71 | 0.06 |
|  |  |  |  | 2013 | 0.19 | 0.05 |
| Dropped terms |  |  |  |  |  |  |
|  |  |  |  | Singleton | -2.87 | 2.40 |
| Growth*Twin | 1 | <0.01 | 0.95 | Singleton vs Twin | -0.03 | 0.19 |
| Growth*Year | 3 | 3.39 | 0.34 | 2010 | -2.85 | 2.36 |
| 2011 |  |  |  | 2010 vs 2011 | 1.00 | 2.65 |
| 2012 |  |  |  | 2010 vs 2012 | -1.58 | 3.17 |
| 2013 |  |  |  | 2010 vs 2013 | 2.84 | 2.56 |
|  |  |  |  | Female | 1.74 | 1.32 |
| Growth*Sex | 1 | 1.57 | 0.21 | Female vs Male | 2.08 | 1.69 |
| Twin | 1 | 0.51 | 0.47 |  | -0.04 | 0.05 |
| Growth | 1 | 0.19 | 0.66 |  | -0.40 | 0.92 |
| Sex | 1 | 0.67 | 0.41 |  | 0.03 | 0.05 |
| Random effect | Standard deviation | | Variance |  |  |  |
| Maternal identity | <0.01 | | <0.01 |  |  |  |
| Residual | 0.25 | | 0.06 |  |  |  |
| **(B)** Malondialdehyde | | |  |  |  |  |
| Final model  (conditional R^2^= 0.44) | | | |  |  |  |
| Year | **3** | **49.07** | **<0.001** | Intercept | 1.68 | 0.08 |
|  |  |  |  | 2011 | 0.12 | 0.09 |
|  |  |  |  | 2012 | -0.38 | 0.10 |
|  |  |  |  | 2013 | -0.25 | 0.09 |
| Dropped terms |  |  |  |  |  |  |
|  |  |  |  | Singleton | 3.00 | 4.28 |
| Growth*Twin | 1 | 0.02 | 0.89 | Singleton vs Twin | 0.55 | 4.23 |
| Growth*Year | 3 | 1.04 | 0.79 | 2010 | 3.09 | 4.21 |
| 2011 |  |  |  | 2010 vs 2011 | -3.67 | 4.78 |
| 2012 |  |  |  | 2010 vs 2012 | -0.66 | 5.50 |
| 2013 |  |  |  | 2010 vs 2013 | -0.52 | 4.65 |
|  |  |  |  | Female | 1.45 | 2.30 |
| Growth*Sex | 1 | 0.29 | 0.59 | Female vs Male | 1.47 | 2.78 |
| Sex | 1 | <0.01 | 0.99 |  | <-0.01 | 0.06 |
| Twin | 1 | 0.49 | 0.49 |  | -0.06 | 0.09 |
| Growth | 1 | 3.05 | 0.08 |  | 2.62 | 1.49 |
| Random effect | Standard deviation | | Variance |  |  |  |
| Maternal Identity | 0.26 | | 0.07 |  |  |  |
| Residual | 0.37 | | 0.14 |  |  |  |
| **(C)** Superoxide dismutase | | |  |  |  |  |
| Final model  (conditional R^2^= 0.64) | | | |  |  |  |
| Year | **3** | **108.79** | **<0.001** | Intercept | 4.23 | 0.61 |
|  |  |  |  | 2011 | 7.65 | 0.71 |
|  |  |  |  | 2012 | 4.07 | 0.76 |
|  |  |  |  | 2013 | 3.49 | 0.70 |
| Dropped terms |  |  |  |  |  |  |
| Growth*Year | 3 | 2.81 | 0.42 | 2010 | 29.42 | 32.36 |
| 2011 |  |  |  | 2010 vs 2011 | -51.97 | 36.21 |
| 2012 |  |  |  | 2010 vs 2012 | -53.59 | 41.21 |
| 2013 |  |  |  | 2010 vs 2013 | -54.24 | 35.33 |
|  |  |  |  | Singleton | -14.33 | 18.07 |
| Growth*Twin | 1 | 1.11 | 0.29 | Singleton vs Twin | -33.00 | 31.48 |
|  |  |  |  | Female | -19.50 | 17.32 |
| Growth*Sex | 1 | 1.77 | 0.18 | Female vs Male | 27.41 | 20.73 |
| Growth | 1 | 0.17 | 0.68 |  | -5.73 | 13.91 |
| Sex | 1 | 0.12 | 0.73 |  | 0.14 | 0.41 |
| Twin | 1 | 0.55 | 0.46 |  | -0.44 | 0.60 |
| Random effect | Standard deviation | | Variance |  |  |  |
| Maternal identity | 2.32 | | 5.37 |  |  |  |
| Residual | 2.58 | | 6.67 |  |  |  |
| **(D)** Total antioxidant capacity | | |  |  |  |  |
| Final model |  |  |  |  |  |  |
| *None* |  |  |  |  |  |  |
| Dropped terms |  |  |  |  |  |  |
|  |  |  |  | Singleton | -5.45 | 10.62 |
| Growth*Twin | 1 | 0.03 | 0.88 | Singleton vs Twin | -1.61 | 10.53 |
| Growth*Year | 3 | 2.02 | 0.57 | 2010 | -5.71 | 10.45 |
| 2011 |  |  |  | 2010 vs 2011 | -3.50 | 11.78 |
| 2012 |  |  |  | 2010 vs 2012 | -3.41 | 14.00 |
| 2013 |  |  |  | 2010 vs 2013 | -13.09 | 11.44 |
|  |  |  |  | Female | -11.62 | 5.81 |
| Growth*Sex | 1 | 2.28 | 0.13 | Female vs Male | 10.91 | 7.34 |
| Sex | 1 | 0.03 | 0.87 |  | 0.03 | 0.16 |
| Twin | 1 | 0.57 | 0.45 |  | -0.16 | 0.22 |
| Growth | 1 | 1.62 | 0.20 |  | -4.77 | 3.75 |
| Year | 3 | 6.26 | 0.10 |  |  |  |
| 2011 |  |  |  |  | 0.14 | 0.24 |
| 2012 |  |  |  |  | 0.17 | 0.26 |
| 2013 |  |  |  |  | 0.44 | 0.23 |
| Random effect | Standard deviation | | Variance |  |  |  |
| Maternal identity |  | 0.35 | 0.13 |  |  |  |
| Residual |  | 1.06 | 1.13 |  |  |  |

Table 2
