# Supplementary material for: Marker-dependent associations among oxidative stress, growth and survival during early life in a wild mammal
Source: Proc Biol Sci. 2016 Oct 12;283(1840):20161407. doi: 10.1098/rspb.2016.1407 (PMC5069507; doi:10.1098/rspb.2016.1407)
Supplement: Appendix Table S3 [file rspb20161407supp5.docx]

**Table S3.** Linear mixed effect model of MDA in response to growth rate (kg day^-1^) in Soay sheep lambs and associated degrees of freedom (DF), estimates and standard errors. The intercept was set to year= 2010 and sex= female. The model shows terms which remained significant after model simplification, along with terms dropped from the model during the simplification process (in order of elimination). The model includes samples collected in August of 2007 and 2010-2013 from male and female lambs (n= 320).

| Term | DF | X^2^ | p-value | Fixed effects | Estimate | Standard error |
| --- | --- | --- | --- | --- | --- | --- |
| **2007** MDA included |  |  |  |  |  |  |
| Final model  (conditional R^2^= 0.31) | | | |  |  |  |
| Year | **4** | **77.96** | **<0.001** | Intercept | 1.47 | 0.12 |
|  |  |  |  | 2007 | -0.40 | 0.09 |
|  |  |  |  | 2011 | 0.13 | 0.09 |
|  |  |  |  | 2012 | -0.44 | 0.10 |
|  |  |  |  | 2013 | -0.29 | 0.09 |
| Growth | **1** | **4.92** | **0.03** |  | 2.58 | 1.17 |
| Dropped terms |  |  |  |  |  |  |
|  |  |  |  | Singleton | 3.37 | 3.79 |
| Growth*Twin | 1 | <0.01 | 0.96 | Singleton vs Twin | -0.18 | 3.45 |
| Growth*Year | 4 | 1.61 | 0.81 | 2010 | 3.33 | 3.72 |
| 2007 |  |  |  | 2010 vs 2007 | -0.62 | 4.23 |
| 2011 |  |  |  | 2010 vs 2011 | -4.02 | 4.29 |
| 2012 |  |  |  | 2010 vs 2012 | -0.43 | 5.10 |
| 2013 |  |  |  | 2010 vs 2013 | -0.77 | 4.17 |
|  |  |  |  | Female | 1.74 | 1.86 |
| Growth*Sex | 1 | 0.21 | 0.65 | Female vs Male | 1.04 | 2.31 |
| Sex | 1 | 0.03 | 0.85 |  | -0.01 | 0.05 |
| Twin | 1 | 0.61 | 0.43 |  | -0.05 | 0.07 |
| Random effect | Standard deviation | | Variance |  |  |  |
| Maternal Identity | 0.14 | | 0.02 |  |  |  |
| Residual | 0.38 | | 0.14 |  |  |  |
