# Supplementary material for: Marker-dependent associations among oxidative stress, growth and survival during early life in a wild mammal
Source: Proc Biol Sci. 2016 Oct 12;283(1840):20161407. doi: 10.1098/rspb.2016.1407 (PMC5069507; doi:10.1098/rspb.2016.1407)
Supplement: Appendix Table S4 [file rspb20161407supp6.docx]

**Table S4.** Generalized linear mixed model of first winter survival as a function of four different biomarkers of oxidative stress (protein carbonyls (PC), malondialdehyde (MDA), superoxide dismutase (SOD) and total antioxidant capacity (TAC)) in Soay sheep lambs and associated degrees of freedom (DF), estimates and standard errors. The intercept was set to female lambs in 2010. All models show terms retained after model simplification, along with dropped terms in order of elimination. The model used data from 135 lambs collected in 2010 and 2013.

| Term | DF | X^2^ | p-value | Fixed effects | Estimate | Standard error |
| --- | --- | --- | --- | --- | --- | --- |
| Survival |  |  |  |  |  |  |
| Final model  (conditional R^2^= 0.54) | | | | Intercept | -6.82 | 2.63 |
| SOD | **1** | **4.30** | **0.04** | SOD | 0.16 | 0.09 |
| August weight | **1** | **15.32** | **<0.001** | August weight | 0.42 | 0.17 |
| PC*Sex | **1** | **5.74** | **0.02** | PC*Sex | -6.02 | 3.12 |
| Sex |  |  |  | Sex | 1.24 | 1.30 |
| PC |  |  |  | PC | -0.23 | 1.56 |
| Dropped terms |  |  |  |  |  |  |
| MDA*Year | 1 | 0.01 | 0.92 |  | -0.11 | 1.06 |
| SOD*Sex | 1 | 0.09 | 0.77 |  | 0.05 | 0.17 |
| PC*Year | 1 | 0.28 | 0.60 |  | 1.64 | 3.30 |
| MDA*Sex | 1 | 0.25 | 0.62 |  | 0.60 | 1.06 |
| TAC*Year | 1 | 0.71 | 0.40 |  | 0.51 | 0.70 |
| SOD*Year | 1 | 0.80 | 0.37 |  | 0.18 | 0.22 |
| TAC*Sex | 1 | 1.79 | 0.18 |  | 0.49 | 0.40 |
| MDA | 1 | 1.04 | 0.31 |  | -0.56 | 0.52 |
| TAC | 1 | 0.98 | 0.32 |  | 0.17 | 0.18 |
| Year | 1 | 1.66 | 0.20 |  | -0.83 | 0.70 |
| Random effect | Standard deviation | | Variance |  |  |  |
| Maternal identity | 0.94 | | 0.88 |  |  |  |
